# A patient with gastric cancer with peritoneal carcinomatosis treated with intraperitoneal chemotherapy who survived more than 5 years receiving repeated laparoscopic examinations: a case report

**DOI:** 10.1186/s13256-016-0799-5

**Published:** 2016-01-19

**Authors:** Hironori Yamaguchi, Joji Kitayama, Hironori Ishigami, Shigenobu Emoto, Takeshi Nishikawa, Junichiro Tanaka, Toshiaki Tanaka, Tomomichi Kiyomatsu, Kazushige Kawai, Keisuke Hata, Hiroaki Nozawa, Shinsuke Kazama, Soichiro Ishihara, Eiji Sunami, Toshiaki Watanabe

**Affiliations:** 1 Department of Surgical Oncology, The University of Tokyo, 7-3-1 Bunkyo-ku, Hongo, Tokyo, 113-8655 Japan; 2 Department of Chemotherapy, The University of Tokyo, 7-3-1 Bunkyo-ku, Hongo, Tokyo, 113-8655 Japan

**Keywords:** Laparoscopy, Peritoneal carcinomatosis, Gastric cancer, Intraperitoneal infusions, Paclitaxel

## Abstract

**Background:**

Peritoneal dissemination of gastric cancer is still a dismal disease and has extremely poor prognosis even with systemic intensive chemotherapy. However, intraperitoneal chemotherapy using paclitaxel has recently shown good results. In order to perform optimal intraperitoneal chemotherapy, laparoscopic examination is necessary to assess the condition of peritoneal disseminated lesions. This is the first report of a case of a patient with gastric cancer with massive peritoneal metastasis treated with intraperitoneal administration of paclitaxel and repeated laparoscopic examinations who survived more than 5 years.

**Case presentation:**

Here we report a case of a 60-year-old Japanese woman with peritoneal carcinomatosis of gastric cancer who underwent intraperitoneal chemotherapy receiving repeated laparoscopic examinations. The patient was referred to our institution for the treatment of peritoneal carcinomatosis of gastric cancer. The staging laparoscopy showed peritoneal metastasis in the whole peritoneal space with a peritoneal cancer index score of 23. An intraperitoneal access port was subcutaneously implanted. Paclitaxel was intraperitoneally and intravenously administered with oral administration of S-1. The second-look laparoscopy, which was performed after nine courses of intraperitoneal chemotherapy, revealed the disappearance of peritoneal carcinomatosis. A total gastrectomy with D2 lymphadenectomy was performed and intraperitoneal chemotherapy was continued after the surgery. The third laparoscopic examination, which was performed after 67 courses of intraperitoneal chemotherapy showed bilateral ovarian metastasis without recurrence of peritoneal carcinomatosis. Since multiple bone metastases developed after the third-look laparoscopy, bilateral adnexectomy was not performed and the chemotherapy was changed to the regimen including CPT-11. Our patient survived more than 5 years since the intraperitoneal chemotherapy started.

**Conclusions:**

Sequential intraperitoneal chemotherapy could strongly suppress the development of peritoneal metastasis for several years. Repeated laparoscopic examinations are considered to be essential to evaluate the efficacy of intraperitoneal chemotherapy on peritoneal carcinomatosis of gastric cancer.

## Background

Peritoneal metastasis of gastric cancer is still a dismal disease and has extremely poor prognosis even with systemic intensive chemotherapy. We have treated advanced gastric cancer patients with peritoneal metastasis by intravenous (IV) and intraperitoneal (IP) administration of paclitaxel (PTX) with oral intake of S-1 [1–4]. In this IP chemotherapy, PTX is repeatedly administered into the intraperitoneal space via a subcutaneously placed intraperitoneal access port with a catheter, the tip of which is placed in the intraabdominal space. This combination chemotherapy is remarkably effective for peritoneal lesions and appears to produce a marked prolongation of survival of those patients [2–4].

Since it is difficult to precisely evaluate peritoneal metastatic lesions with imaging modalities such as computed tomography (CT) or magnetic resonance imaging (MRI), laparoscopic examinations are necessary to assess the condition of intraperitoneal lesions. Here we report the case of a patient with gastric cancer with massive peritoneal carcinomatosis who survived more than 5 years with intraperitoneal chemotherapy and repeated laparoscopic examinations.

## Case presentation

A 60-year old Japanese woman was diagnosed with gastric cancer at another hospital. A staging laparoscopic examination was performed at that hospital and peritoneal metastasis was diagnosed. The patient was referred to our hospital to receive IP chemotherapy for peritoneal carcinomatosis of gastric cancer.

**Fig. 1:**
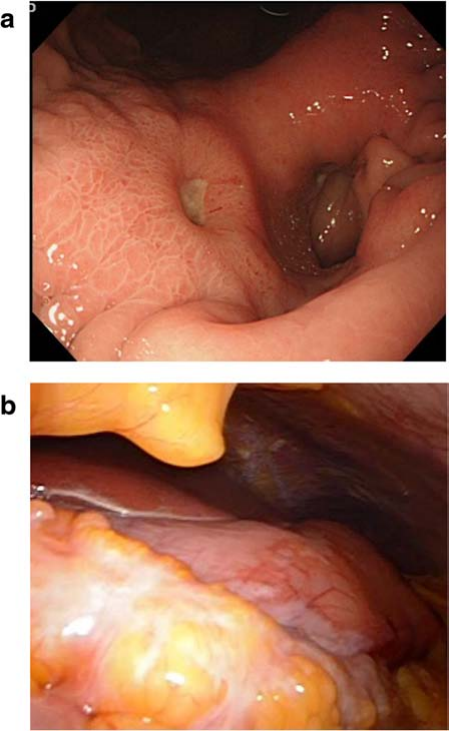
**a** Scirrhus-type gastric cancer with ulcer. **b** Staging laparoscopy revealed serosal invasion of primary gastric cancer and peritoneal carcinomatosis involving omentum

**Fig. 2:**
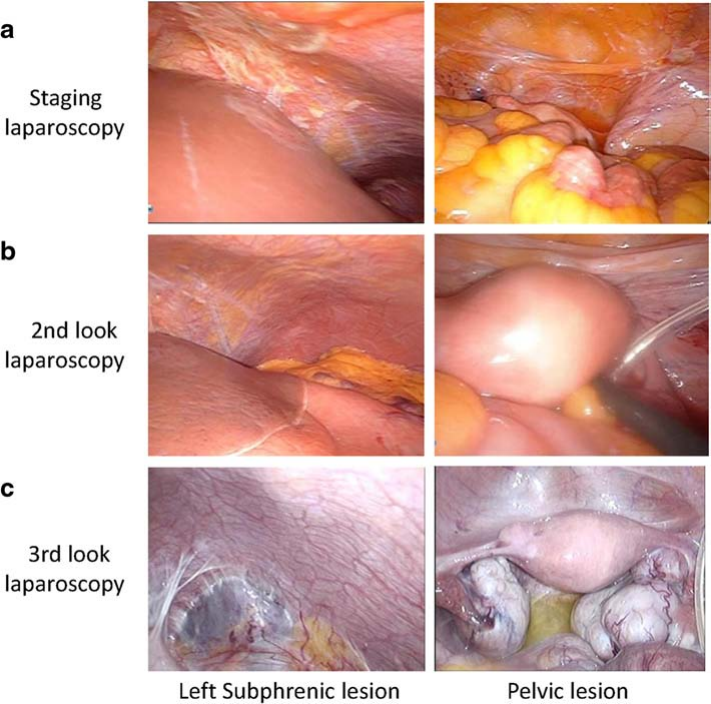
**a** Staging laparoscopy revealed peritoneal dissemination of gastric cancer in the whole intraperitoneal space. **b** Second-look laparoscopy showed the disappearance of peritoneal carcinomatosis after intraperitoneal chemotherapy. **c** Third-look laparoscopy showed sustained disappearance of peritoneal carcinomatosis but development of bilateral ovarian metastasis 4 years and 6 months after intraperitoneal chemotherapy started

The upper endoscopy showed scirrhous-type gastric cancer (Fig. 1a) with the biopsy result of moderately to poorly differentiated adenocarcinoma. A staging laparoscopy was performed also in our institution showing the tumor exposure to serosa of the stomach and the massive peritoneal carcinomatosis involving the greater omentum (Fig. 1b). Peritoneal disseminated nodules were found in whole intraabdominal space with a peritoneal cancer index (PCI) score of 23 (Fig. 2a). Intraperitoneal washing fluid cytology was positive for free cancer cells. An IP access port was placed in the subcutaneous space.

The regimen of the intraperitoneal chemotherapy is oral intake of S-1 for 14 days, IP PTX (20 mg/kg) and IV PTX (50 mg/kg) on day 1 and day 8 with 7 days’ rest. This treatment was performed as a clinical study approved by the institutional review board of the University of Tokyo. After our patient received nine courses of chemotherapy in 7 months, a second-look laparoscopy was performed to assess the effect of the intraperitoneal chemotherapy. The disseminated peritoneal nodules had disappeared (Fig. 2b) and the washing cytology results had turned negative.

After confirming the effectiveness of intraperitoneal chemotherapy, a total gastrectomy with D2 lymph node dissection was performed. Three of 14 harvested lymph nodes were found to have metastasis. The pathological grading of regression was 2.

After gastrectomy, our patient received the same regimen of intraperitoneal chemotherapy. A follow-up CT scan, which was performed 4 years and 3 months after the IP chemotherapy started, revealed bilateral ovary enlargement. In order to reevaluate the conditions of the peritoneal disseminated lesions, a third-look laparoscopic examination was performed after our patient underwent a total of 67 courses of intraperitoneal chemotherapy in 4 years and 6 months, which revealed that the peritoneal carcinomatosis had not recurred. However, bilateral enlarged ovaries were observed, which were considered to be Krukenberg metastasis (Fig. 2c).

A bilateral adnexectomy was planned but not actually performed because bone scintigraphy revealed multiple bone metastases. The chemotherapy was then changed to the regimen including CPT-11. After the IP chemotherapy was stopped, the peritoneal carcinomatosis recurred. Our patient survived 5 years and 2 months after her diagnosis of peritoneal carcinomatosis of gastric cancer.

## Conclusions

In this case, the peritoneal disseminated lesions remained suppressed by continuous IP administration of paclitaxel for at least 4 years and 6 months. This clinical course clearly shows the strong suppression effect of sequential IP chemotherapy on peritoneal carcinomatosis of gastric cancer. The condition of the peritoneal disseminated lesions could not be assessed by CT or MRI. The optimal treatment strategy for patients with peritoneal carcinomatosis who have received IP chemotherapy can be decided appropriately by performing repeated laparoscopic examinations.

## Consent

Written informed consent was obtained from the son of the patient for publication of this case report and any accompanying images. A copy of the written consent is available for review by the Editor-in-Chief of this journal.

## Abbreviations

CT: Computed tomography
IP: Intraperitoneal
IV: Intravenous
MRI: Magnetic resonance imaging
PCI: Peritoneal cancer index
PTX: Paclitaxel
